# A Modified Reverse Remplissage Procedure for Management of a Locked Posterior Shoulder Dislocation

**DOI:** 10.1155/2020/8625368

**Published:** 2020-05-28

**Authors:** Xavier Zwiebel, Stéphane Pelet, Alexandre Leclerc

**Affiliations:** ^1^Université Laval, Pavillon Ferdinand-Vandry, 1050 Avenue de la Médecine, Quebec City, QC, Canada G1V 0A6; ^2^Department of Orthopaedic Surgery, CHU de Québec–Hopital de l'Enfant-Jésus, 1401, 18ème Rue, Quebec City, QC, Canada G1J 1Z4; ^3^Department of Orthopaedic Surgery, CHU de Québec–Centre Hospitalier de l'Université Laval (CHUL), 2705 Laurier Boulevard, Quebec City, QC, Canada G1V 4G2

## Abstract

Posterior shoulder dislocation is rare and often represents a diagnostic and therapeutic challenge. An impaction fracture of the anteroinferior aspect of the humeral head (called a reverse Hill-Sachs (RHS) fracture) is always present in case of chronic locked posterior dislocation. Surgical management is required and decided on the delay between the trauma and the diagnosis and the importance of the RHS (in percentage). The authors present a chronic locked posterior shoulder dislocation in a 32-year-old active male with a reverse Hill-Sachs lesion of more than 40%. An open reduction was required, and stabilization was achieved with a modified remplissage technique with detachment of the upper quarter of the subscapularis tendon. Three years after the surgery, the patient recovered an excellent functional level with a stable shoulder.

## 1. Introduction

Described by Sir Astley Cooper in 1938, traumatic posterior dislocations of the shoulder are an uncommon diagnosis and a challenging clinical problem [1]. These injuries account for up to 5% of shoulder dislocation and are caused by high-energy trauma, seizure, or electrocution. The initial diagnosis is missed or delayed by treating physicians in up to 79% of cases [1–4]. Although multiple reasons can explain this delay, it is more commonly the lack of appropriate radiologic examination [5]. In most cases, the posterior edge of the glenoid causes impaction of the anteromedial aspect of the humeral head. This is known as a reverse Hill-Sachs lesion (RHS). An axillary view or a CT scan is essential in establishing the diagnosis and determining the size of the humeral head defect [5].

Posterior instability (i.e., recurrent posterior dislocations) often requires surgical stabilization. Soft-tissue procedures are preferred (arthroscopic posterior labrum repair, reverse remplissage) and more complex techniques reserved for significant bone defect (McLaughlin procedure, bone grafting, and posterior bone block). Chronic locked posterior shoulder dislocation (CLPSD) is described as a posterior dislocation discovered at least two weeks after the initial event that is not reducible with closed methods. A RHS is always present in patients with CLPSD, and the final treatment is based on its importance. This include disimpaction, autogenous bone grafting of the defect, osteoarticular allograft, lesser tuberosity transfer, subscapularis tendon transfer, or shoulder arthroplasty [1–11].

We present the case of a young active patient with a CLPSD treated with a modified open remplissage technique with detachment of the upper quarter of the subscapularis tendon.

### 1.1. Case Report

**Figure 1:**
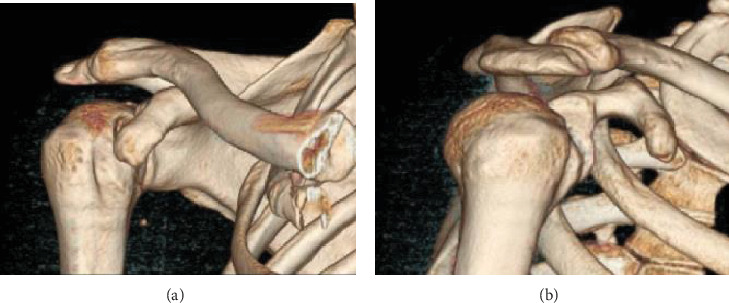
3D CT reconstruction of an AP (a) and lateral view of the shoulder (b).

A 32-year-old military patient was addressed to our clinic 6 weeks after sustaining a closed posterior dislocation of the right shoulder. This resulted from a conducted electrical weapon shot during a military training. The dislocation was recognized but not reduced adequately, and the patient had started rehabilitation. The patient is right-handed. He presented with a painful shoulder with limited motion in all directions (anterior elevation 70°, abduction 50°, and external rotation -10°). Standard shoulder radiographs demonstrated a locked posterior dislocation (Figure 1).

**Figure 2:**
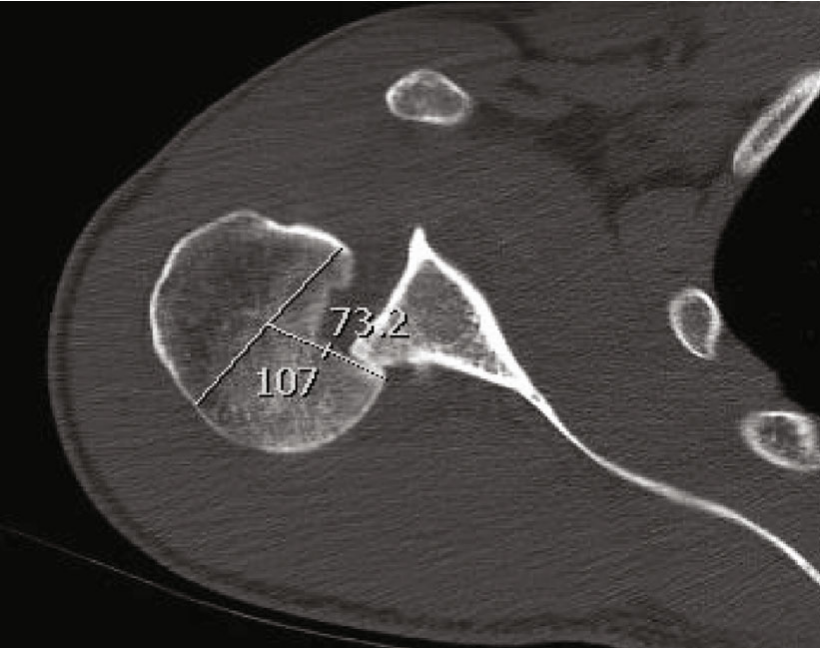
Reverse Hill-Sachs lesion of more than 40%.

A computed tomography scan confirmed the presence of a large RHS lesion of more than 40% (Figure 2).

**Figure 3:**
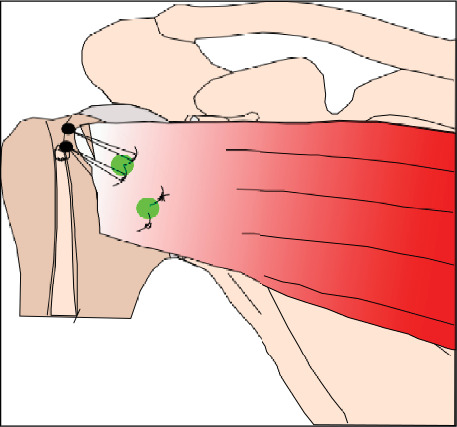
Operative technique.

As the delay between the trauma and the surgery was significant, we decided not to attempt a closed reduction, mainly to prevent a humeral head fracture. Under general anaesthesia in a beach chair position, an open reduction was achieved through a deltopectoral approach. Release of the rotator interval and the upper quarter of the subscapularis tendon was done to ease exposure and reduction was achieved using a blunt instrument along the glenoid and the humeral head. The RHS lesion engaged at less than 20-degree internal rotation in neutral abduction. The RHS lesion was debrided to stimulate tissue healing, and two BioComposite Corkscrew 5.5 mm anchors (Arthrex, Naples, Florida, USA) were fixed in the cavity: the first in the inferior and medial part and the second in the upper and more posterior part. The upper quarter of the subscapularis was slightly released from the anterior glenoid rim; then, the eight strands were passed through the subscapularis tendon and tied to reproduce a remplissage technique. The upper quarter of the subscapularis was repaired with a double-row technique using two Swivel Lock anchors 5.5 mm (Arthrex, Naples, Florida, USA) within the bicipital groove. The final insertion of the subscapularis tendon is oblique with a small lengthening of the upper part and a small shortening of the inferior part (Figure 3). The long head of the biceps was pathological (fraying), and a tenodesis was performed in the inferior part of the bicipital groove, using the remaining sutures in the Swivel Lock anchor. The shoulder was stable in internal rotation, and there was no excessive restriction in external rotation.

The patient's shoulder was maintained in an abduction/external (30°/10°) rotation brace for 6 weeks; then, physical therapy was initiated. The patient returned to his full activities (military and sports) 6 months after the surgery without any limitation.

**Figure 4:**
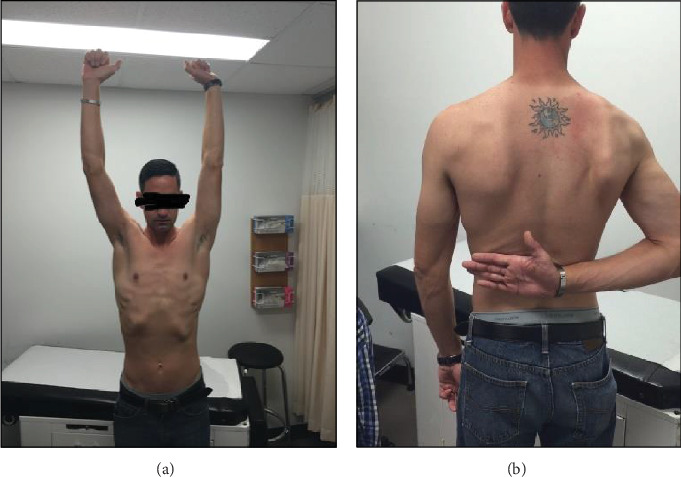
Final range of motion in abduction and anterior elevation (a) and internal rotation (b).

The last follow-up was performed three years after the surgery. The patient presented with a painless shoulder (during daily living activities and work), and no recurrences occur. He is fully secure with his shoulder and does not complain of any apprehension. The active range of motion of the shoulder is very good and quite similar to the contralateral shoulder: anterior elevation 150 (vs. 180), abduction 160 (vs. 180), external rotation with arm at side 60 (vs. 80), and internal rotation at T12 (vs. T8) (Figure 4). The shoulder was stable in all directions (no anterior or posterior apprehension, symmetric anterior and posterior drawer test, and negative load-and-shift test). The overall abduction strength (measured with a dynamometer) demonstrate a slight deficit in isometric (14%) and isokinetic (11.3%) strength compared to the contralateral shoulder. Descriptive common functional scores were recorded and qualified as good: Western Ontario Shoulder Instability Index (WOSI) 18.3%, Oxford Shoulder Score (OSS) 34, American Shoulder Elbow Surgeon (ASES) 63.3, and Melbourne Instability Shoulder Scale (MISS) 82%.

**Figure 5:**
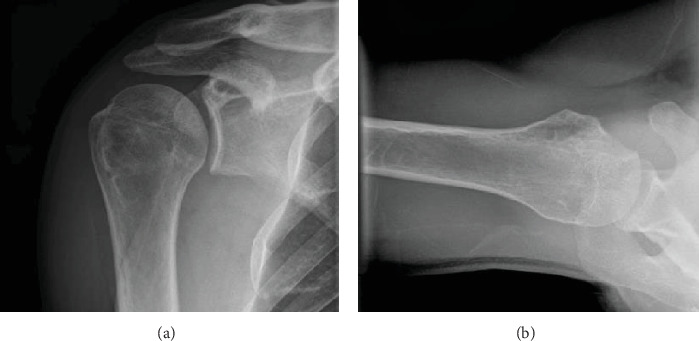
Postoperative AP (a) and (b) axillary views of the shoulder.

Final radiographs show a congruent reduced shoulder (Figure 5).

## 2. Discussion

Posterior shoulder dislocations are rare injuries. The most common mechanism is trauma, such as direct blow to the humeral head, fall on an outstretched arm, or motor vehicle collision [12, 13]. It is also often secondary to epileptic seizure or electrocution. A CT scan is essential to determine the treatment strategy by quantifying the size of humeral head defect and identifying associated fractures, as observed in 50% of cases [3, 12, 14].

Treatment options and strategies range from conservative treatment to total shoulder arthroplasty [1–3, 6, 10–13]. There is no clear consensus in the current literature. Small case series provide some guidance, principally based on the duration of the dislocation, the size of the Hill-Sachs lesion, and the patient condition [15, 16].

We presented the case of a young, high-demand military male, suffering a locked chronic posterior shoulder dislocation with a massive reverse Hill-Sachs lesion. The late presentation prevented a closed reduction that could be associated with a high risk of fracture, worsening of the humeral head defect, or head necrosis [11]. In order to restore the best functional shoulder with slight limitation, surgical options are limited.

Allograft or autograft reconstruction of the RHS is proposed for major humeral head defect but tends to have higher reoperation rates and complications such as head necrosis and graft resorption. These options also required a more aggressive dissection with complete opening of the subscapularis tendon [7, 15, 17]. Arthroplasty (hemi or total) is a valuable option in cases of major reverse Hill-Sachs lesion in older or low-demand patients. Functional results are interesting but not as good as arthroplasty for primary glenohumeral arthritis and should not be the first option in young active patients [18, 19]. Some authors recently described an arthroscopic reverse remplissage technique with the subscapularis tendon without its disinsertion [20–22] or with the medial glenohumeral ligament (MGHL) [23]. These techniques require no major dissection and are more respectful of the anatomy but invariably shorten the subscapularis and lead to a loss of external rotation. Arthroscopic reverse remplissage is proposed for posterior instability (or acute reducible posterior dislocation) with small RHS involving less than one-third of the articular surface. This technique was not possible in this case.

Subscapularis transfer described by McLaughlin and its recent modifications demonstrated good clinical results when the RHS lesion is less or around 30-40% of the humeral head [8, 11–13, 15–19]. The partial or complete tendon transfer (with or without the lesser tuberosity) results in a significant restriction of range of motion, mainly in abduction and external rotation. There is sometimes a need for a second surgery, either to remove implants or to decompress the subcoracoid space. The surgical exposition provided would be interesting to perform a safe open reduction of a locked posterior shoulder, but the anticipated functional limitations led us to develop an alternative procedure for a young active patient.

The modified remplissage technique used in this case is easy and reproducible. At our knowledge, this is the first report of this modified technique. Compared to standard techniques involving the subscapularis tendon, the benefits are a perfect exposure to reduce a locked shoulder, no significant shortening of the subscapularis tendon (only the inferior part), only little reduction in abduction and external rotation, and no need for further surgeries (like subcoracoid space decompression or hardware removal). This constitutes a good alternative between arthroscopic and large open procedures and avoids jeopardizing the anatomy for potential future surgical treatments. This should be reserved for very specific patients with a chronic posterior locked shoulder (even with large RHS lesion) and high functional expectations.

## 3. Conclusion

The modified remplissage technique is an interesting alternative for chronic locked posterior shoulder dislocation, even with a large RHS lesion. This technique is more anatomic and provided an excellent long-term functional result in a young active patient. This should be reserved for very specific conditions as described in this report.
